# Trichina Spiralis

**Published:** 1868-09

**Authors:** 


### TRICHINA SPIRALIS.

In the next number of the REGISTER we commence the publication of a translation by Prof. R. King Browne, of a condensed and very comprehensive treatise upon "Trichina Spiralis," embracing its history, production, peculiarities, its mode of action, and the results, with illustrations.

This is, probably, the best paper extant upon this subject, and its first appearance in the English language. It will certainly receive marked consideration from those interested in this subject. And who is not? It is not one intimately connected with Dental practice, but it is desirable for the Dentist to have a large range of knowledge of pathological conditions. This treatise gives a very clear description of an affection that is generally very imperfectly understood. T.

